# Diet and Muscle Metabolism

**DOI:** 10.3390/nu17101727

**Published:** 2025-05-20

**Authors:** Domenico Azzolino, Vincenzo Gianturco, Riccardo Calvani

**Affiliations:** 1 Geriatric Unit, Fondazione IRCCS Ca’ Granda Ospedale Maggiore Policlinico di Milano, 20122 Milan, Italy; 2 Department of Geriatrics, Orthopedics and Rheumatology, Università Cattolica del Sacro Cuore, L.go F. Vito 1, 00168 Rome, Italy; riccardo.calvani@unicatt.it; 3 Fondazione Policlinico Universitario “Agostino Gemelli” IRCCS, L.go A. Gemelli 8, 00168 Rome, Italy

Advancing age is associated with a progressive loss of muscle mass and strength/function, termed *sarcopenia*, which leads to a wide spectrum of adverse outcomes including falls, institutionalization, loss of independence, and mortality [1]. Muscle decline starts after the age of 40 and intensifies later in life, where the loss of muscle mass and function reaches a rate of 0.6–1% and 3% per year, respectively [2]. Sarcopenia is a prototypical geriatric condition. Perturbations at multiple levels, from cellular processes within myocytes to systemic factors (i.e., hormonal changes and chronic inflammation), as well as social and environmental determinants, may contribute to its development [3]. This Special Issue on “Diet and Muscle Metabolism” has been conceived to include studies aimed at (1) providing an overview of the State of the Art in the field; (2) exploring the possible biological mechanisms underlying musculoskeletal abnormalities; and (3) enhancing our current understanding regarding potential areas of intervention.

The studies published in this Special Issue investigated the physiological and metabolic dimensions of sarcopenia and its related phenotypes, with a focus on the effects of dietary intake, metabolic flexibility, sex, and clinical interventions on aging muscle. Despite the differences in the methodologies employed, which ranged from animal models to interventional clinical trials, these investigations share several key characteristics that enhance the current understanding of muscle health throughout life.

Alterations in most biological hallmarks of aging have been described in older adults with sarcopenia [4]. Advancing age is accompanied by a state of chronic, low-grade inflammation, the so-called “inflamm-aging” [5]. This phenomenon is characterized by altered circulating concentrations of pro- and anti-inflammatory cytokines (including the “usual suspects” interleukin (IL) 1, IL-6, and tumor necrosis factor-alpha (TNF-α)), and may lead to reductions in food intake, metabolic changes (i.e., the elevation of resting energy expenditure), and increased muscle catabolism [6]. Mitochondrial dysfunction in skeletal myocytes is another major determinant of age-related muscle atrophy [7]. Mitochondria are the major cellular source of reactive oxygen species (ROS) and play a critical role in both metabolic and quality control processes in skeletal myocytes [8]. Alterations in mitochondrial function led to the overproduction of ROS, which directly damage intracellular macromolecules (i.e., proteins, lipids, and nucleic acids). Furthermore, excessive ROS production stimulates the release of mitochondrial-derived damage-associated molecular patterns (DAMPs), such as mtDNA and mitochondrial formylated peptides, which stimulate the innate immune system response and promote a persistent inflammatory milieu. The consequent secretion of inflammatory mediators may, in turn, promote further mitochondrial damage, thus creating a vicious cycle [9].

Skeletal muscle also undergoes profound structural changes with aging [10]. Besides myofiber atrophy and loss, intermuscular fat infiltration affects muscle strength and mobility function via lipotoxic effects. Excessive adipose tissue may further induce the release of pro-inflammatory mediators, which enhances muscle loss [11] and promotes the development of sarcopenic obesity, a condition associated with higher morbidity and mortality in older adults [12]. In this Special Issue, Moroni et al. [13], using a large cohort of 1510 Italian adults, reported that sarcopenia was the most prevalent phenotype (17%), followed by osteosarcopenia (14.7%) and sarcopenic obesity (2%). The study also identified several biochemical markers linked to these conditions, such as inflammatory (C-reactive proteins and erythrocyte sedimentation rate) and nutritional biomarkers (albumin and iron), underscoring the interplay between chronic inflammation, nutrient status, and musculoskeletal health in aging populations.

Gut dysbiosis, i.e., the imbalance in the gut microbiota composition and function, is emerging as another factor that influences age-related muscle decline. Gut dysbiosis is associated with increased intestinal permeability, which favors the passage of endotoxins and other microbial factors, thereby eliciting a systemic inflammatory response [14]. Fatigue has been proposed as an early clinical indicator of muscle metabolism abnormalities [15]. When the dietary intake of energy and protein is not adequate to meet individual demands, body fat and muscles are catabolized to provide energy substrates [16,17,18]. This leads to metabolic dyshomeostasis, which is associated with fatigue onset [19]. Both mitochondrial quality and quantity are reduced in the presence of fatigue [20]. Impairments in mitochondrial numbers, biogenesis, and activity, which are common features of muscle wasting diseases, including sarcopenia, seem to be the main cellular mechanisms underlying the development of fatigue in older adults [20].

Three studies published in this Special Issue explored the role of different dietary patterns in shaping muscle health and metabolic function in animal models. Alameddine et al. [21] demonstrated that a maternal low-protein diet during lactation led to reduced muscle mass and strength in offspring, with effects more pronounced in males. While female offspring initially recovered muscle weight after weaning, they experienced significant muscle loss during aging. Da Eira et al. [22] compared the effects of a high-fat, sucrose-enriched (HFS) diet and a carbohydrate-free ketogenic diet (KD) on skeletal muscle metabolism in rats. Although both increased fat availability, only the KD preserved insulin-stimulated glucose metabolism, improved mitochondrial markers, and promoted muscle-type-specific ketone utilization. In contrast, the HFS diet impaired metabolic flexibility. Hulett et al. [23] investigated sex-specific skeletal muscle adaptations to a high-fat, high-sucrose (HFHS) diet, showing that male rats exhibited elevated mitochondrial respiration and insulin resistance, despite similar fat gain between sexes. Transcriptomic analyses highlighted distinct sex-dependent differences in nutrient handling and signaling pathways, including PI3K/AKT and PPARα/RXRα. Collectively, these studies emphasize the critical influence of diet on muscle health and metabolic function, underscore sex-specific responses, and support the need for personalized nutritional strategies to prevent or manage obesity, metabolic disorders, and muscle-related conditions.

Although several drug candidates are under investigation, no pharmacological treatments are currently available to counteract age-related muscle decline [24]. The main strategies to prevent and manage sarcopenia are based on lifestyle interventions (i.e., diet and physical activity) [25]. Among these, resistance training and a protein-rich diet/supplementation have been reported to be the most effective in counteracting muscle decline [26].

In this Special Issue, Rondanelli et al. [27] reported that a two-month multidisciplinary residential program (MRP) significantly improved key health metrics in institutionalized adults with sarcopenic obesity. The MRP included a personalized low-energy mixed diet, exercise sessions (e.g., aerobic plus resistance training) five days a week, and cognitive behavioral therapy. Participants demonstrated enhanced physical performance, as indicated by increased Short Physical Performance Battery (SPPB) scores, alongside favorable changes in body composition, including reductions in fat mass and visceral adipose tissue. Moreover, the intervention led to marked improvements in glycemic control, lipid profiles, and insulin sensitivity. These findings highlight the effectiveness of comprehensive, supervised interventions in mitigating the clinical burden of sarcopenic obesity.

Other dietary compounds like omega-3 polyunsaturated fatty acids (PUFAs), vitamin D, creatine, and β-hydroxy-β-methylbutyrate (HMB) have shown promising results in preserving muscle mass in older adults. Recent systematic reviews and meta-analyses reported the positive effects of PUFA supplementation on muscle strength and function, with controversial results for lean mass [28]. Low levels of vitamin D have been associated with reduced muscle strength and function in longitudinal studies [29,30]. However, meta-analyses of vitamin D supplementation’s effects on muscle parameters reported mixed results, especially when it was considered as a stand-alone therapy [31,32,33]. Creatine supplementation has been suggested as a promising strategy for counteracting muscle atrophy due to its role in muscle protein synthesis and satellite cell activation, mediated by the insulin-like growth factor 1–mammalian target of rapamycin axis [34]. Finally, HMB, a metabolite of leucine, has been found to stimulate muscle anabolism in institutionalized older patients [35]. However, recent systematic reviews reported only minor effects of HMB supplementation in attenuating age-related muscle mass loss in older adults, while the results on strength/function were inconclusive [36].

A wide array of nutraceuticals, including resveratrol [37], quercetin [38], ursolic acid [39], urolithin [40], fisetin [41], and nicotinamide riboside [42,43], are being increasingly studied for their potential effects on critical myocyte pathways, such as autophagy, cellular senescence, and mitochondrial biogenesis [44]. However, although pre-clinical studies have shown promising results, the beneficial effects of these biomolecules on human muscle aging still remain to be proven.

Over the last few decades, various research findings have answered many questions about age-related musculoskeletal conditions. However, given their complex and multifactorial etiology, a deeper understanding of the biological mechanisms underlying alterations in skeletal muscle metabolism is key for developing new effective and targeted strategies to preserve muscle mass and function/strength in advanced age. The optimization of the current approaches through personalized nutritional interventions represents a promising strategy to combat age-related muscle loss. The development of new pharmacological agents may be of particular interest among individuals not responding to lifestyle interventions, or for those in whom they cannot be implemented.
